# Bempegaldesleukin (BEMPEG; NKTR‐214) efficacy as a single agent and in combination with checkpoint‐inhibitor therapy in mouse models of osteosarcoma

**DOI:** 10.1002/ijc.33382

**Published:** 2020-11-25

**Authors:** Marlene Hennessy, Andrew Wahba, Kumar Felix, Mariella Cabrera, Maria Gabriela Segura, Vikas Kundra, Murali K. Ravoori, John Stewart, Eugenie S. Kleinerman, Vanessa Behrana Jensen, Vidya Gopalakrishnan, Rhoneil Pena, Phi Quach, Grace Kim, Saul Kivimäe, Loui Madakamutil, Willem W. Overwijk, Jonathan Zalevsky, Nancy Gordon

**Affiliations:** ^1^ Nektar Therapeutics San Francisco California USA; ^2^ Children's Memorial Hermann Hospital UT Health Science Center Houston Texas USA; ^3^ Department of Pharmaceutical Sciences Hampton University Hampton Virginia USA; ^4^ Department of Pediatrics Lincoln Medical and Mental Health Center New York New York USA; ^5^ Division of Pediatrics, Department of Pediatrics Research The University of Texas MD Anderson Cancer Center Houston Texas USA; ^6^ Verge Genomics South San Francisco California USA; ^7^ Invivoscribe San Diego CA USA

**Keywords:** bempegaldesleukin, checkpoint inhibitors, IL‐2 receptor agonist, NKTR‐214, osteosarcoma

## Abstract

Survival of patients with relapsed/refractory osteosarcoma has not improved in the last 30 years. Several immunotherapeutic approaches have shown benefit in murine osteosarcoma models, including the anti‐programmed death‐1 (anti‐PD‐1) and anti‐cytotoxic T‐lymphocyte antigen‐4 (anti‐CTLA‐4) immune checkpoint inhibitors. Treatment with the T‐cell growth factor interleukin‐2 (IL‐2) has shown some clinical benefit but has limitations due to poor tolerability. Therefore, we evaluated the efficacy of bempegaldesleukin (BEMPEG; NKTR‐214), a first‐in‐class CD122‐preferential IL‐2 pathway agonist, alone and in combination with anti‐PD‐1 or anti‐CTLA‐4 immune checkpoint inhibitors in metastatic and orthotopic murine models of osteosarcoma. Treatment with BEMPEG delayed tumor growth and increased overall survival of mice with K7M2‐WT osteosarcoma pulmonary metastases. BEMPEG also inhibited primary tumor growth and metastatic relapse in lungs and bone in the K7M3 orthotopic osteosarcoma mouse model. In addition, it enhanced therapeutic activity of anti‐CTLA‐4 and anti‐PD‐1 checkpoint blockade in the DLM8 subcutaneous murine osteosarcoma model. Finally, BEMPEG strongly increased accumulation of intratumoral effector T cells and natural killer cells, but not T‐regulatory cells, resulting in improved effector:inhibitory cell ratios. Collectively, these data in multiple murine models of osteosarcoma provide a path toward clinical evaluation of BEMPEG‐based regimens in human osteosarcoma.

## Abstract 2

**What's new?**

Despite treatment, many patients with osteosarcoma are at high risk for metastatic disease. Such cases of advanced osteosarcoma present significant therapeutic challenges, owing largely to a lack of novel and optimized treatment strategies. Here, the authors investigated the efficacy of bempegaldesleukin (BEMPEG; NKTR‐214), a novel CD122‐preferential interleukin‐2 pathway agonist, in murine models of osteosarcoma. Experiments show that BEMPEG monotherapy decreases metastatic relapse in lung and bone tissue and prolongs survival in mice. Durable tumor growth was achieved with combined BEMPEG and anti‐CTLA‐4 or anti‐PD‐1 checkpoint inhibition. The data provide a scientific rationale for clinical evaluation of BEMPEG for osteosarcoma treatment.

**Abbreviations**
BEMPEG: bempegaldesleukin (NKTR‐214)
CTLA‐4: cytotoxic T‐lymphocyte antigen‐4
DMEM: Dulbecco's modified Eagle's medium
IHC: immunohistochemistry
IL‐2: interleukin‐2
NK: natural killer
PBS: phosphate‐buffered saline
PD‐1: programmed death‐1
PEG: polyethylene glycol
rhIL‐2: recombinant human interleukin‐2
TGI: tumor growth inhibition
Treg: regulatory T cells

## 1 INTRODUCTION

Osteosarcoma is the most common primary malignant bone tumor.^1^ Primary osteosarcoma occurs predominantly in adolescents and young adults under the age of 25 years, while it generally presents as a secondary malignancy in adults over 65 years of age.^2^, ^3^ Along with surgical resection, combination chemotherapy plays a central role in the management of osteosarcoma; however, a lack of new therapies and the inability to optimize existing ones has prevented significant improvement in overall survival for the last 30 years. Moreover, treated patients remain at high risk for developing pulmonary metastases, the leading cause of death from osteosarcoma.^4^ Thus, relapsed/refractory osteosarcoma remains challenging to treat and constitutes an unmet clinical need.^5^, ^6^, ^7^, ^8^, ^9^, ^10^

Several novel immunotherapeutic approaches have been evaluated for the treatment of sarcomas, including osteosarcoma, such as monoclonal antibodies directed against the immune checkpoint protein targets cytotoxic T‐lymphocyte antigen‐4 (CTLA‐4) and programmed death‐1 (PD‐1).^1^, ^11^, ^12^, ^13^, ^14^ These immune checkpoint inhibitors (ICI) modulate the adaptive immune response by preventing the downregulation of T‐cell function induced by immune checkpoints.^15^ In recent years, ICIs have induced high response rates, particularly in “hot tumors” that have high immune infiltrates, including melanoma and lung cancer.^16^ In “cold tumors” with low infiltrating T cells, such as osteosarcoma, the benefit of ICIs is less clear.^16^ In preclinical murine models of osteosarcoma, when used alone or in combination with other therapies, antibodies against CTLA‐4 or PD‐1 have shown benefit by upregulating the antitumor activity of cytotoxic T cells.^11^, ^12^, ^13^ In a xenograft osteosarcoma mouse model, anti‐PD‐1 therapy resulted in regression of pulmonary metastases; such efficacy was mediated by local M1‐polarized macrophages.^4^ However, in clinical studies of osteosarcoma, ICIs have demonstrated limited clinical activity.^17^, ^18^, ^19^ Nevertheless, a recent immunogenomic study using multiplatform profiling of osteosarcoma specimens from 48 pediatric and adult patients with primary, relapsed and metastatic osteosarcoma confirmed multiple immunosuppressive features of osteosarcoma, suggesting potential immunotherapeutic opportunities.^19^ Thus, it is thought that novel therapeutic approaches that stimulate T cells may synergize with ICIs to induce antitumor responses.

High‐dose systemic recombinant human interleukin‐2 (rhIL‐2) is an immunotherapeutic approach with efficacy in melanoma and renal cell carcinoma (RCC), likely through stimulating the proliferation and antitumor activity of T cells and natural killer (NK) cells. Although some efficacy has also been observed in a limited group of pediatric patients with sarcoma, high toxicity rates, poor pharmacokinetics and the pleiotropic effects of systemic rhIL‐2 have precluded its further development as a viable therapeutic option.^20^, ^21^, ^22^ To improve efficacy while minimizing toxicity, alternate formulations of IL‐2 have been developed, such as aerosols that can directly target immune cells in the lungs. Preclinical studies of aerosolized rhIL‐2, both as a single agent and in combination with adoptive transfer of NK and/or T cells, have demonstrated efficacy in osteosarcoma.^23^, ^24^, ^25^

Recently, novel forms of IL‐2 have been engineered to improve efficacy and decrease toxicity relative to rhIL‐2. Bempegaldesleukin (also known as BEMPEG or NKTR‐214) is a CD122‐preferential IL‐2‐pathway agonist that is being investigated for its ability to stimulate an antitumor response via induction of T‐effector cells.^26^ It consists of an rhIL‐2 moiety conjugated to an average of six releasable polyethylene glycol (PEG) chains.^27^ When BEMPEG is administered systemically, the PEG chains are progressively released from rhIL‐2, unmasking its activity as an IL‐2 receptor agonist with preferential IL‐2 receptor beta (CD122) binding in a pharmacokinetically predictable, controlled and sustained manner.^27^, ^28^, ^29^ Compared to native IL‐2, the placement of the PEG chains directs BEMPEG to preferentially bind the heterodimeric IL‐2 receptor beta gamma complex (IL‐2Rβγ; CD122/CD132), most frequently expressed on T‐effector cells, over the heterotrimeric IL‐2Rαβγ complex, typically expressed on Tregs.^27^, ^28^, ^29^ BEMPEG favors activation and expansion of T‐effector cells and NK cells, without expansion of unwanted regulatory CD4^+^ T cells (Treg) in the tumor tissue.^27^, ^28^, ^29^ Mechanistically, the lack of Treg expansion observed in the tumor microenvironment is mediated by CD8^+^ T‐cell‐associated cytokines, including IFNγ and TNFα, which are released in response to cognate tumor antigen recognition in the tumor tissue.^28^ BEMPEG treatment has resulted in stronger antitumor activity with better tolerability than that with rhIL‐2 in a variety of mouse models, when given either alone or in combination with anti‐CTLA‐4, anti‐PD‐1, adoptive T‐cell therapy or cancer vaccines.^27^, ^28^, ^29^, ^30^ Early clinical data revealed that BEMPEG was well tolerated and strongly increased numbers of peripheral and intratumoral T cells and NK cells, and promoted a favorable intratumoral ratio of effector T cells to Tregs.^26^ Combination therapy with BEMPEG and anti‐PD‐1 (nivolumab) showed efficacy in multiple cancer types^31^ and received U.S. Food & Drug Administration Breakthrough Therapy Designation for first‐line treatment for patients with metastatic melanoma. Based on these results, we evaluated the efficacy of BEMPEG, alone and in combination with anti‐PD‐1 and/or anti‐CTLA‐4 ICIs in metastatic and orthotopic murine models of osteosarcoma.

## 2 MATERIALS AND METHODS

### 2.1 Reagents and cell lines

BEMPEG was provided by Nektar Therapeutics (San Francisco, CA). Therapeutic anti‐CTLA‐4 and anti‐PD‐1 monoclonal antibodies were obtained from BioXCell (Lebanon, NH). Labeled antibodies and other reagents for flow cytometry were obtained from eBioscience (San Diego, CA). Cell culture and general laboratory reagents were obtained from Sigma‐Aldrich (St. Louis, MO) and Gibco/Thermo‐Fisher Scientific (Waltham, MA).

The K7M2‐WT (Research Resource Identifier [RRID]: CVCL_V455) murine metastatic osteosarcoma cell line, first described by Khanna and colleagues,^32^ was obtained from American Type Culture Collection (Manassas, VA). The K7M3 (RRID: CVCL_XG68) aggressive metastatic osteosarcoma cell line described by Gordon and colleagues^33^ was originally developed by cycling the K7M2‐WT cell line in mice via tail vein injection and harvesting the resulting lung metastases for further culture. The K7M3 cells were obtained from the Kleinerman's laboratory (MD Anderson Cancer Center, Houston, TX). The DLM8 (RRID: CVCL_6669) cell line was obtained from RIKEN BioResource Center (Ibaraki, Japan).

Cell lines were maintained at 37°C and 5% CO_2_, in sterile tissue culture flasks, with Dulbecco's modified Eagle's medium (DMEM; K7M2‐WT, K7M3) containing nonessential amino acids, sodium pyruvate, l‐glutamine or MEM (DLM8) and 10% fetal bovine serum. The cells were tested routinely and found to be free of mycoplasma (MycoAlert Mycoplasma Kit, Lonza, Allandele, NJ) contamination.

### 2.2 Animal models

Four‐week‐old female BALB/c mice were purchased from the National Cancer Institute (Bethesda, MD), and 5‐ to 6‐week‐old female BALB/c and C3H mice were purchased from Charles River Breeding Laboratories. Animal studies were conducted with the approval of the Institutional Animal Care and Use Committees at the University of Texas MD Anderson Cancer Center and Nektar Therapeutics. Animals were provided standard irradiated diet and water ad libitum. For osteosarcoma models, criteria for removing animals from efficacy studies through humane sacrifice included tumor volume ≥ 2000 mm^3^, tumor ulceration, excessive body weight loss, respiratory distress and neurological symptoms.

#### 2.2.1 Disseminated K7M2‐WT metastatic osteosarcoma mouse model

BALB/c mice were inoculated with 3 × 10^6^ K7M2‐WT cells via lateral tail vein injection. The day after inoculation was designated as treatment Day 0. BEMPEG (0.8 mg/kg) was intravenously administered on Days 0, 9 and 18 in the prophylactic regimen and on days 9, 18 and 27 in the therapeutic regimen. Satellite animals were periodically sacrificed to detect the development of macroscopic metastatic lung lesions, which appeared around treatment Day 9 (initiation of therapeutic treatment regimen). Efficacy studies included 10 mice per treatment group, with survival as the primary study endpoint. Additionally, four mice per group were sacrificed for flow cytometric immune cell profiling on Day 17 in the therapeutic regimen and on Day 21 in the prophylactic regimen.

#### 2.2.2 K7M3 primary tibial osteosarcoma mouse model

BALB/c mice underwent two intratibial inoculations of 1 × 10^5^ cultured K7M3 cells passaged no more than four times. Prior to inoculation, the left rear leg was cleaned with iodine and 70% ethanol. Tumor cell suspension was aspirated into a 1 mL disposable syringe fitted with a 27‐gauge needle. Ten microliters of the single‐cell suspension containing 1 × 10^5^ cells was injected twice. Injections were given 24 hours apart and to the same leg following the same procedure. Inoculated mice were assigned to treatment groups of 10 animals per group and treated with vehicle or BEMPEG (0.8 mg/kg intravenously on Days 11, 20, 29, 38 and 47 after inoculation). Treatment efficacy against the primary tumor was evaluated by weekly X‐rays. All X‐ray image analyses were performed using National Institutes of Health's ImageJ software (available at http://www.rsb.info.nih.gov/ij). For assessing tumor volume, the diameter‐based calculation was computed by measuring the greatest longitudinal diameter (length) and the greatest transverse diameter (width) of the tumors. Diameter‐based measurements were determined using the modified ellipsoidal formula^34^, ^35^ to calculate diameter‐based volume (V) in mm^3^ (tumor volume = ½ [length × width^2^]).

Animals underwent amputation 33 days after inoculation when primary tumor reached 1.5 cm^3^. Tibial tumors were evaluated by histology. All mice were sacrificed 45 to 50 days (7 weeks) after initial K7M3 inoculation, at which time relapse and metastasis were assessed by histology of the femur and lungs. Histological response was determined as follows: measurements were carried out using a microscope equipped with a stage clip and an eyepiece graticule. Areas of tumor necrosis were measured at ×100 magnification, whereas areas of viable tumor were measured at ×40 magnification and converted to ×100. One unit area at ×40 is equivalent to 0.087 mm^2^. Measurements were performed twice and averaged.

#### 2.2.3 DLM8 subcutaneous osteosarcoma mouse model

DLM8 tumors were implanted subcutaneously in the flank of C3H mice. Seven days after implantation, animals were randomized; treatment was initiated at a tumor volume of 106 ± 3 mm^3^ (mean ± SE of the mean) and designated as Day 0. Specific treatments and dosing schedules for each group are summarized in Table 1.

**TABLE 1:** Efficacy of BEMPEG, anti‐CTLA‐4 and anti‐PD‐1 monotherapy and combination therapy in murine DLM8 subcutaneous osteosarcoma^a^

Group	Treatment	Route	Dose (schedule)	TGI, %Δ*T*/Δ*C*, Day 29	Survival at 141 days	Tumor‐free at 141 days
1	Vehicle	i.p.	NA (Days 0, 4, 9, 13, 18)	−	0/10	0/10
2	BEMPEG	i.v.	0.8 mg/kg (Days 0, 9, 18)	26	1/10	0/10
3	Anti‐CTLA‐4	i.p.	200 μg (Days 0, 4) and 100 μg (Days 9, 13, 18)	79 (****)	5/10	2/10
4	Anti‐PD‐1	i.p.	100 μg (Days 0, 4) and 200 μg (Days 9, 13, 18)	57 (**)	3/10	2/10
5	BEMPEG + anti‐CTLA‐4	i.v.	0.8 mg/kg (Days 4, 13, 22)	96 (****)	10/10	6/10
		i.p.	200 μg (Days 0, 4) and 100 μg (Days 9, 13, 18)			
6	BEMPEG + anti‐PD‐1	i.v.	0.8 mg/kg (Days 4, 13, 22)	60 (**)	3/10	0/10
		i.p.	100 μg (Days 0, 4) and 200 μg (Days 9, 13, 18)			
7	Anti‐CTLA‐4 + anti‐PD‐1	i.p.	200 μg (Days 0, 4) and 100 μg (Days 9, 13, 18)	86 (****)	8/10	5/10
		i.p.	100 μg (Days 0, 4) and 200 μg (Days 9, 13, 18)			

Abbreviations: BEMPEG, bempegaldesleukin; CTLA‐4, cytotoxic T‐lymphocyte antigen‐4; i.p., intraperitoneal; i.v., intravenous; NA, not applicable; PD‐1, programmed death‐1; TGI, tumor‐growth inhibition.

^a^ Asterisks **, **** indicate *P* < .01, .0001, respectively; log‐rank test vs vehicle.

Tumor volumes and body weights were monitored two to three times per week up to 141 days posttreatment initiation. Percent tumor growth inhibition (%TGI) was calculated using the following formula: %TGI = 1 − (relative tumor volume [%] of treatment group ÷ relative tumor volume [%] of control group) × 100. Treatment efficacy was analyzed using ordinary one‐way analysis of variance followed by Dunnett's multiple comparison test calculated in Prism (GraphPad, San Diego, CA). Complete response was defined as animals with 100% tumor regression (ie, tumor free).

### 2.3 Immunohistochemistry

The resected lungs were washed in saline, fixed in 10% formalin buffer and embedded in paraffin. Five‐micrometer‐thick tissue sections were deparaffinized in xylene and rehydrated. For the primary bone tumors, tissues were fixed in 10% formalin and then decalcified using ethylenediaminetetraacetic acid for 24 hours before embedding in paraffin. Bone tumor tissue sections were heated at 60°C for 24 hours and then subjected to the same process of deparaffinization and rehydration as the lungs. Antigen retrieval was performed for tissues using vector unmasking solution pH 6 diluted in ddH_2_O (1:10). Sections were blocked using normal blocking serum (Vector Laboratories, Burlingame, CA) diluted in phosphate‐buffered saline (PBS) followed by overnight incubation with primary antibodies against CD4 (1:50) and CD8 (1:50) (eBioscience, Waltman, MA). On Day 2, slides were washed using PBS and incubated with 3% hydrogen peroxide for 30 minutes to block endogenous peroxidase. Signal was amplified using a diluted biotinylated secondary antibody (VECTASTAIN Elite ABC HRP Kit; Vector Laboratories) incubated for 30 minutes with further PBS washes and addition of VECTASTAIN ABC reagent for another 30 minutes. Counterstaining using 3,3′‐diaminobenzidine and hematoxylin was performed followed by image capture (Leica Mycrosystems Inc., San Jose, CA) and quantification using the SimplePCI software (Hamamatsu Photonics). Data were expressed as the ratio of positive staining (brown) to nuclear staining (blue). Sections not exposed to the primary antibody served as negative controls, and normal mouse spleen was used as a positive control. Three slides per group were selected, then three different tumor areas per slide were quantified and averaged, and the mean ratio of positive pixel area to total positive counted nuclei was plotted. Measurements from each field and from each group (control or treatment) were analyzed for statistical significance using the Mann‐Whitney *U* test.

### 2.4 Flow cytometry

Mouse lungs were perfused with cold PBS to remove blood, dissociated manually with a scalpel and incubated in digestion buffer (2.5 mg/mL collagenase II, 2.5 mg/mL collagenase IV and 0.5 mg/mL deoxyribonuclease I) at 37°C for 13 to 15 minutes. Infiltrating leukocytes were isolated by centrifugation of single‐cell suspensions using a 40/80 Percoll gradient and collecting cells at the interface. Isolated leukocytes were stained for cell populations of interest and analyzed with a BD LSR II cell analyzer (Becton Dickinson). Flow cytometry data were analyzed using the FlowJo v10 software (FlowJo, LLC, Ashland, OR) and graphed using Prism 6.

The following leukocyte subsets were analyzed from total live cells: total T lymphocytes (CD45^+^CD3^+^); CD4^+^ T lymphocytes (CD45^+^CD3^+^CD4^+^); CD4^+^ Tregs (CD45^+^CD3^+^CD4^+^CD25^+^FoxP3^+^); CD8^+^ T lymphocytes (CD45^+^CD3^+^CD8^+^); B cells (CD45^+^CD3^−^B220^+^); NK cells (CD45^+^CD3^−^B220^−^CD49b^+^) and monocytes (CD45^+^SSCA^low^CD3^−^B220^−^CD49b^−^CD11b^high^). CD25 served as a functional activation marker of CD4^+^ and CD8^+^ cells, and CD11b as a marker of NK cell maturation and activation.

## 3 RESULTS

### 3.1 BEMPEG monotherapy prolongs survival in a disseminated K7M2‐WT model of metastatic osteosarcoma

**FIGURE 1:**
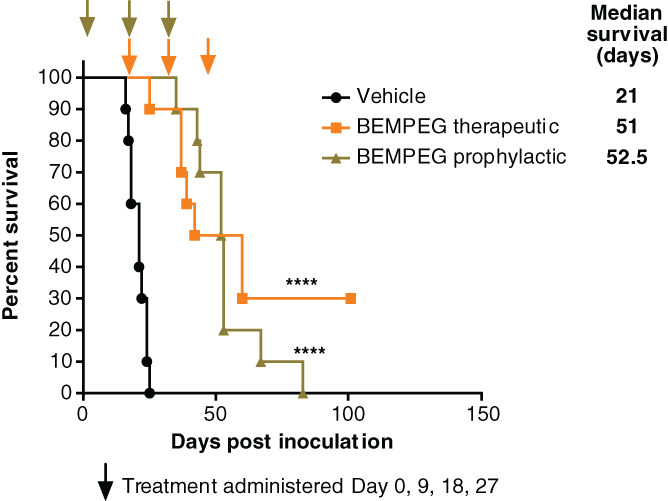
Bempegaldesleukin (BEMPEG; NKTR‐214) effect against K7M2‐WT metastatic osteosarcoma mouse model. Survival analysis in mice inoculated intravenously with K7M2‐WT tumor cells and treated with BEMPEG 0.8 mg/kg (3 doses spaced 9 days apart) or vehicle (black). Arrows indicate treatment days. The prophylactic dosing regimen (green) was initiated on treatment Day 0 (1 day after inoculation) and the therapeutic regimen (orange) was initiated on Day 9. Asterisks (****) indicate *P* < .0001 by log‐rank (Mantel–Cox) [Color figure can be viewed at wileyonlinelibrary.com]

BEMPEG efficacy was tested in mice with disseminated K7M2‐WT syngeneic murine osteosarcoma following prophylactic or therapeutic treatment regimens, as specified in Section 2. Median survival times within the prophylactic and therapeutic BEMPEG groups were significantly higher than in the vehicle group (52.5 and 51 days, respectively, vs 21 days; *P* < .0001, log‐rank [Mantel‐Cox] test; Figure 1). By the last day of study (day 101 after treatment start), 30% of the mice in the therapeutic group were still alive, compared to 0% in the vehicle and prophylactic treatment groups. Both prophylactic and therapeutic BEMPEG regimens demonstrated significant antitumor activity against disseminated K7M2‐WT osteosarcoma.

### 3.2 BEMPEG monotherapy improves the ratio of effector to suppressor immune cell populations in the lungs of mice with pulmonary K7M2‐WT tumors

**FIGURE 2:**
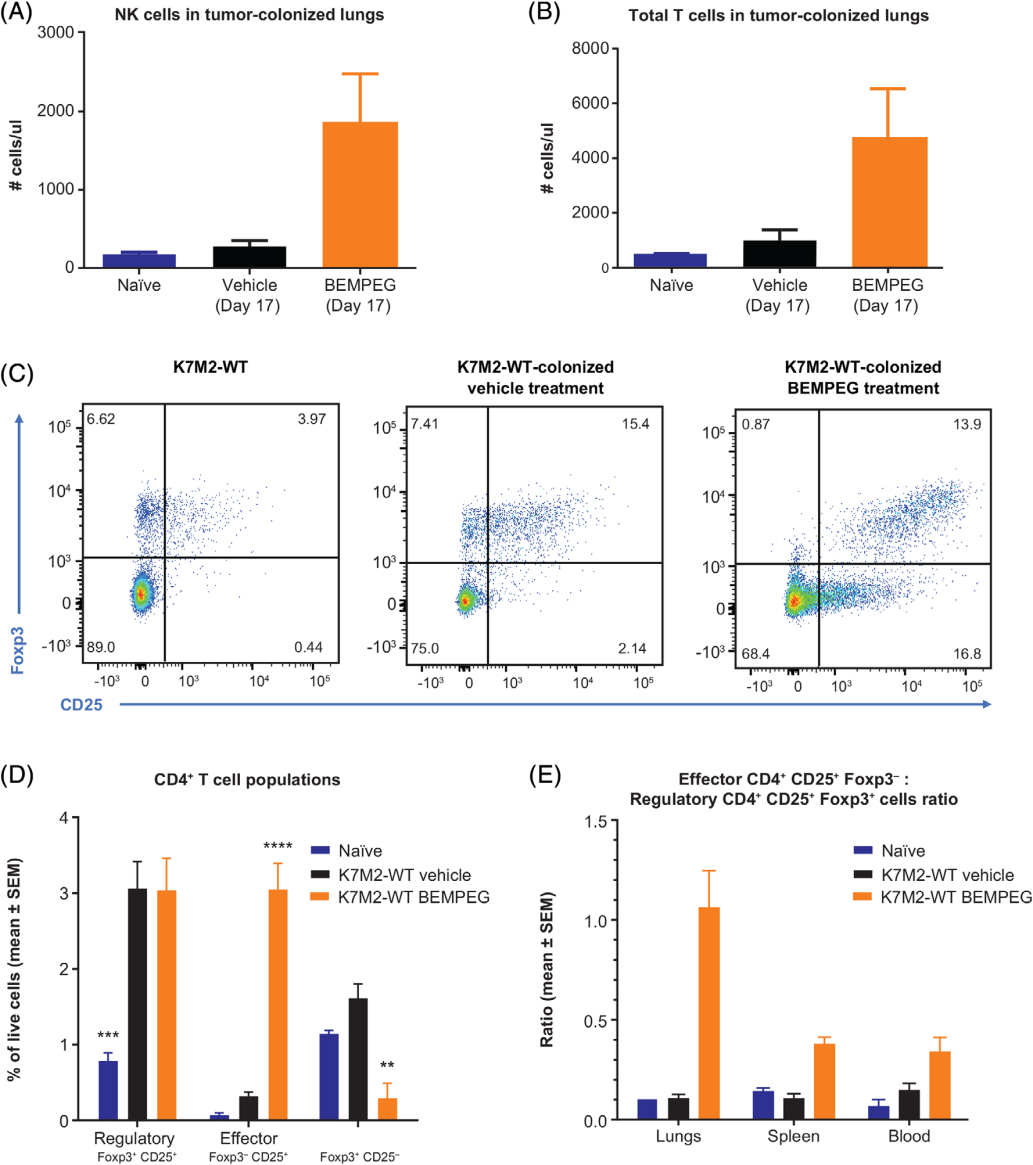
Effect of BEMPEG therapeutic regimen on NK and T‐cell populations in K7M2‐WT tumor‐colonized lungs: A, NK cells in tumor‐colonized lungs on treatment Day 17—therapeutic model; B, total T cells in tumor‐colonized lungs on treatment Day 17—therapeutic model. Increase in CD25 expression and CD4^+^ T effector to CD4^+^ Treg ratio in K7M2‐WT‐colonized mice after BEMPEG treatment, on treatment Day 21—prophylactic model: C, flow cytometry on lymphocytes isolated from K7M2‐WT‐naïve lungs, K7M2‐WT‐colonized lungs treated with vehicle, and K7M2‐WT‐colonized lungs treated with BEMPEG (therapeutic regimen); D, distribution of CD4^+^ T‐cell subpopulations in lungs; E, ratio of T‐effector cells to Tregs in lungs, spleen and whole blood. Asterisks **, ***, **** indicate *P* < .01, .001, .0001, respectively (two‐way ANOVA vs vehicle, Dunnett's posttest). ANOVA, analysis of variance; BEMPEG, bempegaldesleukin; NK, natural killer; Treg, regulatory T cell [Color figure can be viewed at wileyonlinelibrary.com]

Analysis of pulmonary tumors in the K7M2‐WT model 1 week after initiating BEMPEG treatment showed increased infiltration of NK cells and total number of T cells compared to vehicle‐treated mice (Figure 2A,B). In vehicle‐treated mice, there was an increase in myeloid cells, including monocytic cells, with disease progression from Day 1 to Day 17 and a decreased ratio of activated T cells and NK cells to monocytes. This was not observed in lung tumors from mice treated with BEMPEG, and effector cell to myeloid cell ratios more closely resembled those in naïve BALB/c control mice (Supplementary Figure S1).

Tumor implantation caused a dramatic increase in pulmonary CD4^+^CD25^+^Foxp3^+^ Tregs 21 days later, and this increase was unaffected by treatment with BEMPEG (3.1% ± 0.8, 3.1% ± 0.9 and 0.8% ± 0.1 of live cells for vehicle, BEMPEG and no tumor groups, respectively; Figure 2C,D). In contrast, BEMPEG treatment induced the expansion of CD25^+^ Foxp3^−^ effector CD4^+^ T cells (Figure 2C,D), resulting in a 10‐fold increase in the ratio of CD4^+^ effector to CD4^+^ regulatory T‐cell fractions (% of live cells) in the lungs of K7M2‐WT tumor‐bearing mice (1.1 ± 0.2 for BEMPEG vs 0.1 ± 0.0 in the vehicle group; Figure 2E). Systemic BEMPEG treatment‐driven increase in the ratio of CD4^+^ effector to CD4^+^ regulatory T cells was most prominent in the tumor‐bearing lungs. Significantly lower increase in CD4^+^ effector to Treg ratio was observed in the spleen and blood. Notably, CD4^+^ Treg fraction did not change in lungs of treated mice, suggesting that BEMPEG selectively increased the effector CD4^+^ T‐cell fraction at the primary site of metastatic tumor lesion development.

### 3.3 BEMPEG monotherapy decreases K7M3 primary tumor growth in tibia and increases T‐cell infiltration into the tumor

**FIGURE 3:**
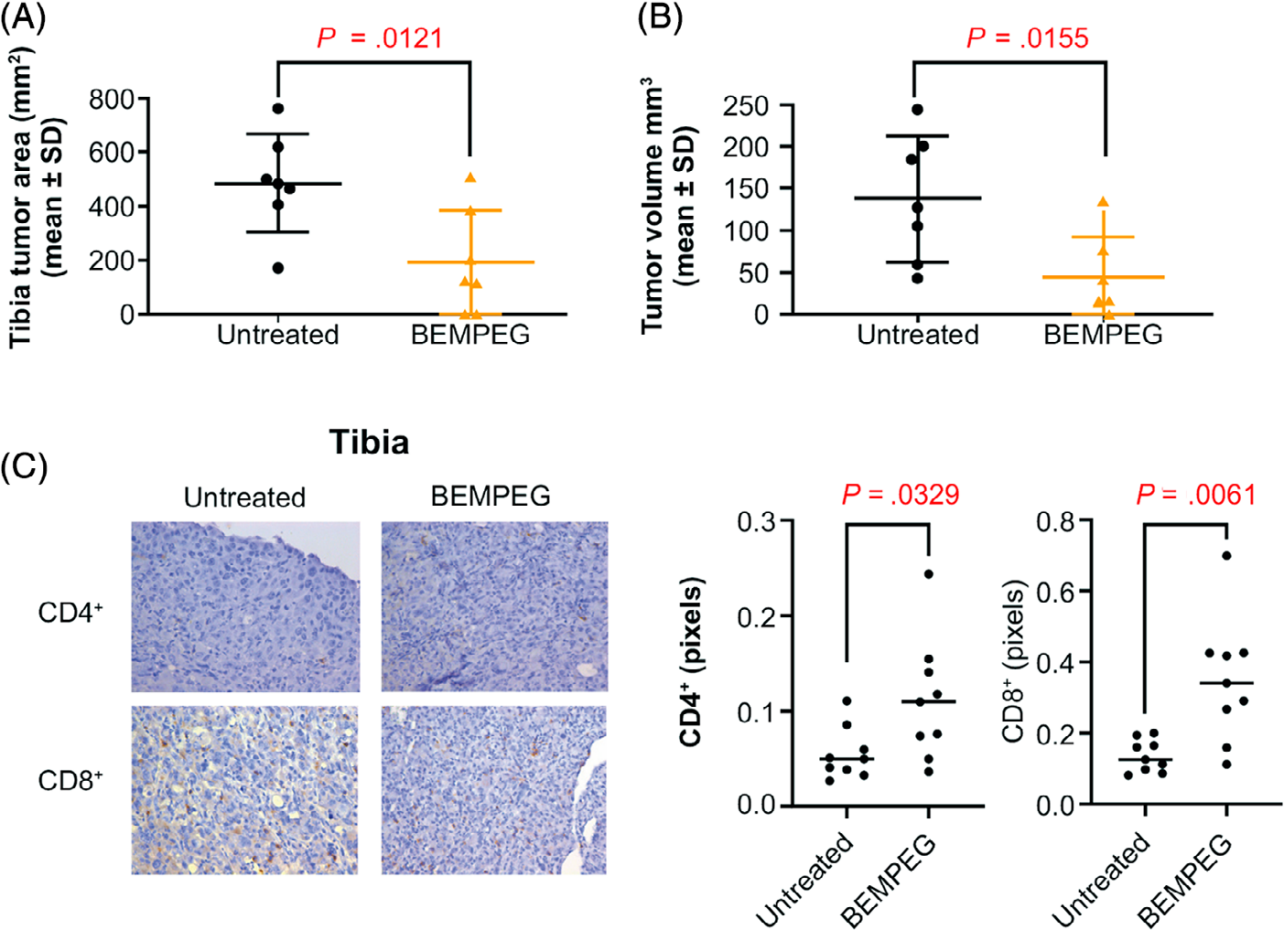
Therapeutic effect of BEMPEG against K7M3 primary osteosarcoma tumor growth. A, Histological analysis at Day 33 postamputation demonstrating a >50% decrease in the tibia tumor area of mice treated with BEMPEG vs untreated control. B, Radiological analysis at Day 33 preamputation demonstrating therapeutic response in the BEMPEG‐treated mice. C, Immunohistochemistry demonstrates an increase in the number of CD8^+^ and CD4^+^ T cells in tumors from mice treated with BEMPEG. BEMPEG, bempegaldesleukin [Color figure can be viewed at wileyonlinelibrary.com]

To examine the effect of BEMPEG on the growth of primary orthotopic osteosarcoma, we performed intratibial injection of K7M3 cells. BEMPEG monotherapy was initiated 11 days after tumor injection. Histological analysis of tibial tumors on Day 33 after amputation showed >50% inhibition of tumor growth as determined by the total tumor area in the BEMPEG‐treated group compared to that in the untreated vehicle control group (190.85 ± 72.47 mm^2^ vs 485.57 ± 68.76 mm^2^, respectively; *P* = .012; Figure 3A). Histological findings were consistent with the radiological analysis of the final primary tumor volume on Day 33 preamputation, for which the mean tumor volume of the BEMPEG‐treated group was about 70% smaller than that of the untreated group (43.14 ± 18.2 mm^3^ vs 137.82 ± 28.21 mm^3^, respectively; *P* = .016; Figure 3B). Decreased primary tibia tumor size was associated with an increase in T‐cell infiltration, as determined by immunohistochemistry (IHC). Mean positive area (pixels) of CD8^+^ T cells (0.3486 vs 0.1358; *P* = .0061) and CD4^+^ (0.1116 vs 0.05519; *P* = .0329) cells was significantly higher in the BEMPEG‐treated group compared with the vehicle control group (Figure 3C). SimplePCI software was used to quantify the IHC staining. Data are expressed as the mean ratio of positive pixel area to total positive nuclei, as described in Section 2.

### 3.4 BEMPEG monotherapy reduces the development of spontaneous K7M3 osteosarcoma lung metastases and increases T‐cell infiltration to the lungs

**FIGURE 4:**
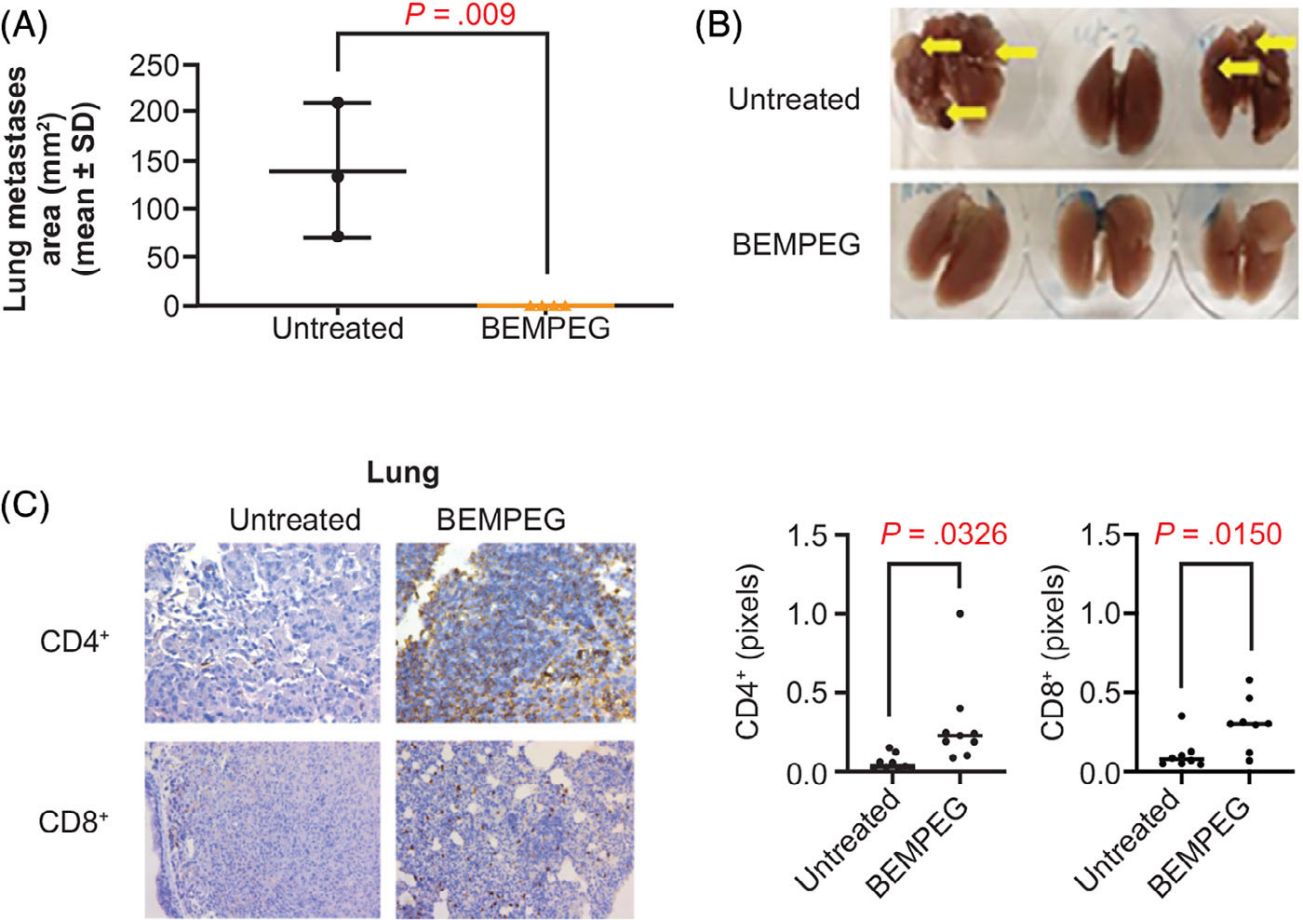
BEMPEG prevents the development of K7M3 osteosarcoma lung metastases. A, Histological analysis of total metastatic lung tumor area in untreated and BEMPEG‐treated lungs demonstrates no evidence of macroscopic lung metastases in the treated group. B, Representative lungs from mice with macroscopic K7M3 osteosarcoma lung metastases untreated and BEMPEG treated. C, Immunohistochemistry staining of CD8^+^ and CD4^+^ T‐cell infiltration into microscopic K7M3 lung tumors demonstrating an increase in immune infiltrates in the BEMPEG‐treated group compared to the untreated control. BEMPEG, bempegaldesleukin [Color figure can be viewed at wileyonlinelibrary.com]

BEMPEG completely prevented spontaneous macroscopic (visible) pulmonary metastasis in mice‐bearing K7M3 primary tibial tumors (0 mm^2^ for BEMPEG vs 139.83 ± 40.36 mm^2^ for vehicle control; *P* = .009; Figure 4A,B). This decrease in lung metastases was associated with a significant increase in T‐cell infiltration by IHC with BEMPEG treatment compared to vehicle control:mean positive area (pixels) of 0.3049 vs 0.1087 (*P* = .0150) for CD8^+^ and 0.2975 vs 0.05684 (*P* = .0326) for CD4^+^ T cells (Figure 4C).

### 3.5 BEMPEG monotherapy decreases osteosarcoma tumor relapse in femurs after amputation of primary tibial tumors

To evaluate local recurrence after surgical resection, the femurs of mice were analyzed 13 days after amputation of the primary tibial K7M3 tumor. All mice (7/7) in the vehicle control group revealed femur colonization, while no tumors (0/7) were found in the femurs of mice treated with BEMPEG (53.7 ± 32.4 mm^3^ vs 0 mm^3^, respectively; *P* = .01; Supplementary Figure S2).

### 3.6 BEMPEG enhances CTLA‐4 monotherapy efficacy on osteosarcoma primary tumor growth inhibition and survival

**FIGURE 5:**
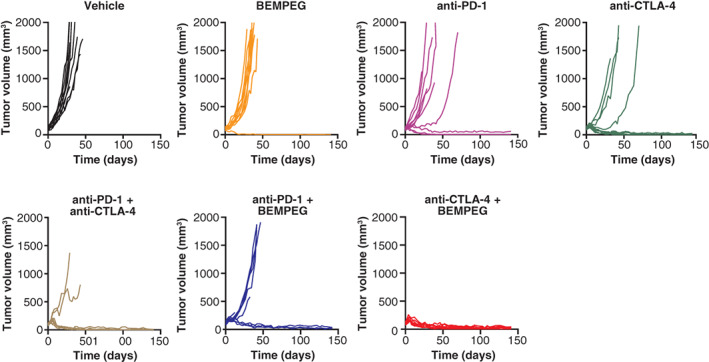
Improvement in the tumor‐growth inhibition activity of checkpoint inhibitors anti‐PD‐1 and anti‐CTLA‐4 when used in combination with BEMPEG, in mice with subcutaneously implanted DLM8 osteosarcoma tumors. BEMPEG, bempegaldesleukin; CTLA‐4, cytotoxic T‐lymphocyte antigen‐4; PD‐1, programmed death‐1 [Color figure can be viewed at wileyonlinelibrary.com]

Anti‐PD‐1 and anti‐CTLA‐4 each suppressed the growth of DLM8 osteosarcoma compared to vehicle control, leading to respective TGI of 57% (*P* < .01) and 79% (*P* < .0001) and 86% (*P* < .0001) for the combination. Addition of BEMPEG to anti‐PD‐1 and anti‐CTLA‐4 resulted in antitumor efficacy of 60% TGI (*P* < .01) and 96% TGI (*P* < .0001), respectively (Table 1). Combination therapy with BEMPEG and anti‐CTLA‐4 was especially effective, preventing significant tumor outgrowth of established primary tumor in 100% of treated animals in the DLM8 model for up to 141 days posttreatment initiation (Figure 5), with 100% surviving animals of which 60% were tumor free (Table 1). Moreover, combination therapy of BEMPEG with anti‐CTLA‐4 provided a significantly greater survival benefit over the 141‐day study duration compared to anti‐CTLA‐4 single agent (*P* = .0157; Supplementary Figure S3).

## 4 DISCUSSION

Osteosarcoma is a difficult disease to treat, with most patients dying of pulmonary metastases. Despite aggressive chemotherapy and successful control of the primary tumor, survival has not improved in the last 30 years; therefore, additional novel therapies are needed. Given the success of ICIs in numerous tumor types, immunotherapy combinations are being explored to optimize treatment by targeting different immunosuppressive mechanisms. For example, ipilimumab plus nivolumab recently demonstrated superior efficacy over standard of care both in patients with metastatic non‐small cell lung cancer (NSCLC) and those with RCC.^36^, ^37^ In our study, murine models of primary and metastatic osteosarcoma were used to study the therapeutic efficacy of BEMPEG both as a monotherapy and in combination with inhibitors of the immune checkpoints PD‐1 and CTLA‐4. Our findings demonstrate preclinical evidence of therapeutic efficacy of BEMPEG alone and in combination with anti‐PD‐1 and anti‐CTLA‐4 in primary and metastatic osteosarcoma.

IL‐2 is a pleiotropic immunomodulatory cytokine that promotes immune tolerance at low doses and robust activation of the antitumor immune response at high doses, inducing proliferation, differentiation and activation of multiple immune cell subsets, notably effector T cells and NK cells.^23^, ^38^, ^39^ In mice‐bearing pulmonary osteosarcoma tumors, targeted delivery of IL‐2 to the lung via aerosol was shown to significantly enhance the therapeutic effect of adoptive transfer of NK cell therapy and, in addition, stimulate local proliferation of NK cells within lung tissue, leading to metastatic control and prolonged survival.^24^, ^25^ Although evidence of IL‐2 efficacy in patients with osteosarcoma is limited, a recent prospective study of 35 unselected pediatric patients treated surgically for primary and metastatic osteosarcoma between 1995 and 2010 revealed promising results.^21^ IL‐2 was administered in addition to high‐dose methotrexate, doxorubicin, cisplatin, ifosfamide and lymphokine‐activated killer cell reinfusion in 27 of 35 patients. The overall and 3‐year event‐free survival rates were 45% and 34%, respectively, suggesting a potential role for IL‐2 in the treatment of osteosarcomas. Another study in relapsed pediatric sarcoma patients found that high‐dose IL‐2 therapy elicited complete responses in two of four osteosarcoma cases.^22^ However, systemic IL‐2 therapy can be difficult to tolerate, primarily due to serious side effects associated with capillary leak syndrome.^23^ Furthermore, its short in vivo half‐life necessitates frequent, repeated dosing. BEMPEG is designed to improve these characteristics through modification of the IL‐2 molecule by conjugation of an average of six PEG chains, which progressively release after administration, creating a long‐acting IL‐2 receptor agonist with preferential IL‐2 receptor beta (CD122) binding.^27^, ^28^, ^29^, ^30^ Early preclinical studies demonstrated superior efficacy of BEMPEG monotherapy to native IL‐2 in an aggressive murine melanoma mouse model. The longer half‐life of BEMPEG resulted in a 500‐fold increase in tumor exposure compared with native IL‐2.^27^ Recent reports describe superior activity of BEMPEG over native IL‐2 in mouse models of adoptive T‐cell transfer and of tumor‐specific vaccination.^28^, ^29^ In patients with advanced solid tumors, BEMPEG monotherapy once every 3 weeks was well tolerated, and strongly increased proliferation and cell numbers of peripheral and intratumoral effector T cells and NK cells without concomitant increase in intratumoral Tregs.^26^ Furthermore, in combination with nivolumab, BEMPEG elicited an objective response rate (ORR) of 60% (n = 22/37; complete response rate: 19%, n = 7/37) in patients with solid tumors, including melanoma, RCC and NSCLC, in the PIVOT‐02 study.^31^ Further data from the dose‐expansion phase of PIVOT‐02 showed an ORR of 53% (n = 20/38; complete response rate: 34%, n = 13/38) in patients with previously untreated metastatic melanoma,^40^ which led to the FDA granting Breakthrough Therapy Designation for BEMPEG plus nivolumab in this indication.

The idea that pulmonary metastasis could conceivably be prevented by activation of immune cells that recognize and kill metastasizing tumor cells was supported with the observation that BEMPEG treatment delayed tumor growth and provided a survival benefit to mice with K7M2‐WT osteosarcoma pulmonary metastases. Lungs of untreated animals showed an increase in immunosuppressive cell populations, including Tregs. Treatment with BEMPEG monotherapy improved the infiltration of effector cells including T cells and NK cells in the tumor‐bearing lungs reversing the effector T cell to Treg ratio in the disease site. A prominent increase in the CD4^+^ effector T‐cell fraction was induced in tumor‐bearing lungs without concomitant changes in Tregs. Such selective increases in effector T cells but not Tregs have also been observed in previously reported nonclinical tumor models.^27^, ^28^ Likewise, patients receiving BEMPEG experienced a strong increase in CD8^+^ effector cells, without expansion of Tregs in the tumor; while Tregs did expand in the periphery (blood).^26^, ^31^ Such tumor tissue‐selective increase in activated effector cells potentially limits cytotoxic activity to disease site and avoids systemic T‐cell activation and nonspecific T‐cell attack in tumor‐free tissues.

In this study, BEMPEG also inhibited primary tumor growth and metastatic relapse in the lungs and bone tissue after K7M3 primary tumor resection, with a concurrent increase in CD8^+^ and CD4^+^ T‐cell infiltration in the bone and lungs. Finally, although BEMPEG monotherapy showed modest efficacy against the DLM8 subcutaneous osteosarcoma model, combination with anti‐CTLA‐4 or anti‐PD‐1 checkpoint inhibitor antibodies led to durable tumor growth control with long‐term survival, including complete cures in a significant fraction of treated animals. Collectively, these data provide a path toward clinical evaluation of BEMPEG‐based regimens in human osteosarcoma.

## CONFLICT OF INTEREST

MH, RP, PQ, GK, SK, LM, WWO and JZ are/were Nektar Therapeutics employees and have/had Nektar Therapeutics stock ownership interests to disclose. All other authors declare that they have no conflicts of interest.

## ETHICS STATEMENT

All studies met the ethical, humane and current regulatory standards for transportation, housing and care established by the American Association of Laboratory Animal Care, the U.S. Department of Agriculture, Department of Health and Human Services and the National Institutes of Health.

## Supporting information


**Supplemental Figure S1** Effect of BEMPEG treatment on ratio of effector (NK and T) cells to monocytes and to SSChi CD11b+/myeloid cells in leukocytes isolated from K7M2‐WT‐colonized lungs. A) NK cells; B) mature (CD11b+) NK cells; C) monocytes; D) NK:monocyte ratio; E) NK:SSChi CD11b + ratio; F) T cell: monocyte ratio
**Supplementary Figure S2**: BEMPEG decreases osteosarcoma relapse to the femur. Representative histological data of mean tumor volume from animals on day 46, 13 days after amputation in the BEMPEG‐treated mice vs untreated controls (*P* = .01).
**Supplementary Figure S3**: Survival of mice subcutaneously implanted with DLM8 osteosarcoma tumors with combination BEMPEG and anti‐CTLA‐4 treatment is higher than anti‐CTLA‐4 alone.

## Data Availability

The data that support the findings of this study are available from the corresponding author upon reasonable request.

## References

[1] Miwa S , Shirai T , Yamamoto N , et al. Current and emerging targets in immunotherapy for osteosarcoma. J Oncol. 2019;2019:7035045. 30693030 10.1155/2019/7035045 PMC6332920

[2] Ottaviani G , Jaffe N . The epidemiology of osteosarcoma. In: Jaffe N , Bruland O , Bielack S , eds. Pediatric and Adolescent Osteosarcoma. Cancer Treatment and Research. Boston, MA: Springer; 2009:3‐13. 10.1007/978-1-4419-0284-9_1 20213383

[3] Casali PG , Bielack S , Abecassis N , et al. Bone sarcomas: ESMO‐PaedCan‐EURACAN clinical practice guidelines for diagnosis, treatment and follow‐up. Ann Oncol. 2018;29:iv79‐iv95. 30285218 10.1093/annonc/mdy310

[4] Dhupkar P , Gordon N , Stewart J , Kleinerman ES . Anti‐PD‐1 therapy redirects macrophages from an M2 to an M1 phenotype inducing regression of OS lung metastases. Cancer Med. 2018;7:2654‐2664. 29733528 10.1002/cam4.1518 PMC6010882

[5] Bielack SS , Kempf‐Bielack B , Delling G , et al. Prognostic factors in high‐grade osteosarcoma of the extremities or trunk: an analysis of 1,702 patients treated on neoadjuvant cooperative osteosarcoma study group protocols. J Clin Oncol. 2002;20:776‐790. 11821461 10.1200/JCO.2002.20.3.776

[6] Geller DS , Gorlick R . Osteosarcoma: a review of diagnosis, management, and treatment strategies. Clin Adv Hematol Oncol. 2010;8:705‐718. 21317869

[7] Khanna C , Fan TM , Gorlick R , et al. Toward a drug development path that targets metastatic progression in osteosarcoma. Clin Cancer Res. 2014;20:4200‐4209. 24803583 10.1158/1078-0432.CCR-13-2574 PMC4134738

[8] Kleinerman E . Maximum benefit of chemotherapy for osteosarcoma achieved‐what are the next steps? Lancet Oncol. 2016;17:1340‐1342. 27569441 10.1016/S1470-2045(16)30270-4

[9] Luetke A , Meyers PA , Lewis I , Juergens H . Osteosarcoma treatment—where do we stand? A state of the art review. Cancer Treat Rev. 2014;40:523‐532. 24345772 10.1016/j.ctrv.2013.11.006

[10] Saraf AJ , Fenger JM , Roberts RD . Osteosarcoma: accelerating progress makes for a hopeful future. Front Oncol. 2018;8:4. 29435436 10.3389/fonc.2018.00004 PMC5790793

[11] Kawano M , Itonaga I , Iwasaki T , Tsumura H . Enhancement of antitumor immunity by combining anti‐cytotoxic T lymphocyte antigen‐4 antibodies and cryotreated tumor lysate‐pulsed dendritic cells in murine osteosarcoma. Oncol Rep. 2013;29:1001‐1006. 23291864 10.3892/or.2013.2224

[12] Lussier DM , O'Neill L , Nieves LM , et al. Enhanced T‐cell immunity to osteosarcoma through antibody blockade of PD‐1/PD‐L1 interactions. J Immunother. 2015;38:96‐106. 25751499 10.1097/CJI.0000000000000065 PMC6426450

[13] Lussier DM , Johnson JL , Hingorani P , Blattman JN . Combination immunotherapy with α‐CTLA‐4 and α‐PD‐L1 antibody blockade prevents immune escape and leads to complete control of metastatic osteosarcoma. J Immunother Cancer. 2015;3:21. 25992292 10.1186/s40425-015-0067-z PMC4437699

[14] Roberts SS , Chou AJ , N‐KV C . Immunotherapy of childhood sarcomas. Front Oncol. 2015;5:181. 26301204 10.3389/fonc.2015.00181 PMC4528283

[15] Dyck L , Mills KHG . Immune checkpoints and their inhibition in cancer and infectious diseases. Eur J Immunol. 2017;47:765‐779. 28393361 10.1002/eji.201646875

[16] Maleki Vareki S . High and low mutational burden tumors versus immunologically hot and cold tumors and response to immune checkpoint inhibitors. J Immunother Cancer. 2018;6:157. 30587233 10.1186/s40425-018-0479-7 PMC6307306

[17] Tawbi HA , Burgess M , Bolejack V , et al. Pembrolizumab in advanced soft‐tissue sarcoma and bone sarcoma (SARC028): a multicentre, two‐cohort, single‐arm, open‐label, phase 2 trial. Lancet Oncol. 2017;18:1493‐1501. 28988646 10.1016/S1470-2045(17)30624-1 PMC7939029

[18] D'Angelo SP , Mahoney MR , van Tine BA , et al. A non‐comparative multi‐center randomized phase II study of nivolumab +/− ipilimumab for patients with metastatic sarcoma (Alliance A091401). Lancet Oncol. 2018;19:416‐426. 29370992 10.1016/S1470-2045(18)30006-8 PMC6126546

[19] Wu C‐C , Beird HC , Andrew Livingston J , et al. Immuno‐genomic landscape of osteosarcoma. Nat Commun. 2020;11:1008. 32081846 10.1038/s41467-020-14646-w PMC7035358

[20] Luksch R , Perotti D , Cefalo G , et al. Immunomodulation in a treatment program including pre‐ and post‐operative interleukin‐2 and chemotherapy for childhood osteosarcoma. Tumori. 2003;89:263‐268. 10.1177/030089160308900306 12908780

[21] Meazza C , Cefalo G , Massimino M , et al. Primary metastatic osteosarcoma: results of a prospective study in children given chemotherapy and interleukin‐2. Med Oncol. 2017;34:191. 29094224 10.1007/s12032-017-1052-9

[22] Schwinger W , Klass V , Benesch M , et al. Feasibility of high‐dose interleukin‐2 in heavily pretreated pediatric cancer patients. Ann Oncol. 2005;16:1199‐1206. 15849223 10.1093/annonc/mdi226

[23] Dhupkar P , Gordon N . Interleukin‐2: old and new approaches to enhance immune‐therapeutic efficacy. Adv Exp Med Biol. 2017;995:33‐51. 28321811 10.1007/978-3-319-53156-4_2

[24] Guma SR , Lee DA , Ling Y , Gordon N , Kleinerman ES . Aerosol interleukin‐2 induces natural killer cell proliferation in the lung and combination therapy improves the survival of mice with osteosarcoma lung metastasis. Pediatr Blood Cancer. 2014;61:1362‐1368. 24610870 10.1002/pbc.25019 PMC4144337

[25] Guma SR , Lee DA , Yu L , et al. Natural killer cell therapy and aerosol interleukin‐2 for the treatment of osteosarcoma lung metastasis. Pediatr Blood Cancer. 2014;61:618‐626. 24136885 10.1002/pbc.24801 PMC4154381

[26] Bentebibel S‐E , Hurwitz ME , Bernatchez C , et al. A first‐in‐human study and biomarker analysis of NKTR‐214, a novel IL2rβγ‐biased cytokine, in patients with advanced or metastatic solid tumors. Cancer Discov. 2019;9:711‐721. 30988166 10.1158/2159-8290.CD-18-1495

[27] Charych DH , Hoch U , Langowski JL , et al. NKTR‐214: an engineered cytokine with biased IL2 receptor binding, increased tumor exposure, and marked efficacy in mouse tumor models. Clin Cancer Res. 2016;22:680‐690. 26832745 10.1158/1078-0432.CCR-15-1631

[28] Sharma M , Khong H , Fa'ak F , et al. Bempegaldesleukin selectively depletes intratumoral Tregs and potentiates T cell‐mediated cancer therapy. Nat Commun. 2020;11:661. 32005826 10.1038/s41467-020-14471-1 PMC6994577

[29] Parisi G , Saco JD , Salazar FB , et al. Persistence of adoptively transferred T cells with a kinetically engineered IL‐2 receptor agonist. Nat Commun. 2020;11:660. 32005809 10.1038/s41467-019-12901-3 PMC6994533

[30] Charych D , Khalili S , Dixit V , et al. Modeling the receptor pharmacology, pharmacokinetics, and pharmacodynamics of NKTR‐214, a kinetically‐controlled interleukin‐2 (IL2) receptor agonist for cancer immunotherapy. PLoS One. 2017;12:e0179431. 28678791 10.1371/journal.pone.0179431 PMC5497954

[31] Diab A , Tannir NM , Bentebibel S‐E , et al. Bempegaldesleukin (NKTR‐214) plus Nivolumab in patients with advanced solid tumors: phase I dose‐escalation study of safety, efficacy, and immune activation (PIVOT‐02). Cancer Discov. 2020;10:1158‐1173. 32439653 10.1158/2159-8290.CD-19-1510

[32] Khanna C , Prehn J , Yeung C , Caylor J , Tsokos M , Helman L . An orthotopic model of murine osteosarcoma with clonally related variants differing in pulmonary metastatic potential. Clin Exp Metastasis. 2000;18:261‐271. 11315100 10.1023/a:1006767007547

[33] Gordon N , Koshkina NV , Jia S‐F , et al. Corruption of the fas pathway delays the pulmonary clearance of murine osteosarcoma cells, enhances their metastatic potential, and reduces the effect of aerosol gemcitabine. Clin Cancer Res. 2007;13:4503‐4510. 17671136 10.1158/1078-0432.CCR-07-0313 PMC4503209

[34] Tomayko MM , Reynolds CP . Determination of subcutaneous tumor size in athymic (nude) mice. Cancer Chemother Pharmacol. 1989;24:148‐154. 2544306 10.1007/BF00300234

[35] Perri GC , Faulk M , Shapiro E , Mellors J , Money WL . Function of the thymus and growth of tumour homograft. Nature. 1963;200:1294‐1296. 14098473 10.1038/2001294a0

[36] Hellmann MD , Paz‐Ares L , Bernabe Caro R , et al. Nivolumab plus ipilimumab in advanced non‐small‐cell lung cancer. N Engl J Med. 2019;381:2020‐2031. 31562796 10.1056/NEJMoa1910231

[37] Motzer RJ , Tannir NM , McDermott DF , et al. Nivolumab plus ipilimumab versus sunitinib in advanced renal‐cell carcinoma. N Engl J Med. 2018;378:1277‐1290. 29562145 10.1056/NEJMoa1712126 PMC5972549

[38] Boyman O , Sprent J . The role of interleukin‐2 during homeostasis and activation of the immune system. Nat Rev Immunol. 2012;12:180‐190. 22343569 10.1038/nri3156

[39] Liao W , Lin J‐X , Leonard WJ . IL‐2 family cytokines: new insights into the complex roles of IL‐2 as a broad regulator of T helper cell differentiation. Curr Opin Immunol. 2011;23:598‐604. 21889323 10.1016/j.coi.2011.08.003 PMC3405730

[40] Diab A , Puzanov I , Maio M , et al. Clinical activity, including deepening of response, of BEMPEG plus NIVO in previously untreated patients with metastatic 1L melanoma: results from the phase 1/2 PIVOT‐02 study. National Harbor, Maryland: Society for Immunotherapy of Cancer; 2019.

