# Supplementary material for: Bempegaldesleukin (BEMPEG; NKTR‐214) efficacy as a single agent and in combination with checkpoint‐inhibitor therapy in mouse models of osteosarcoma
Source: Int J Cancer. 2020 Nov 25;148(8):1928–37. doi: 10.1002/ijc.33382 (PMC7984260; doi:10.1002/ijc.33382)
Supplement: Supplementary file 1 — Supplemental Figure S1 Effect of BEMPEG treatment on ratio of effector (NK and T) cells to monocytes and to SSChi CD11b+/myeloid cells in leukocytes isolated from K7M2‐WT‐colonized lungs. A) NK cells; B) mature (CD11b+) NK cells; C) monocytes; D) NK:monocyte ratio; E) NK:SSChi CD11b + ratio; F) T cell: monocyte ratio Supplementary Figure S2: BEMPEG decreases osteosarcoma relapse to the femur. Representative histological data of mean tumor volume from animals on day 46, 13 days after amputation in the BEMPEG‐treated mice vs untreated controls (P = .01). Supplementary Figure S3: Survival of mice subcutaneously implanted with DLM8 osteosarcoma tumors with combination BEMPEG and anti‐CTLA‐4 treatment is higher than anti‐CTLA‐4 alone. [file IJC-148-1928-s001.pdf]

SUPPLEMENTAL APPENDIX

Bempegaldesleukin (BEMPEG; NKTR-214) efficacy as a single agent and in combination with checkpoint-inhibitor therapy in mouse models of osteosarcoma

Marlene Hennessy, Andrew Wahba, Kumar Felix, Mariella Cabrera, Maria Gabriela Segura, Vikas Kundra, Murali K Ravoori, John Stewart, Eugenie S Kleinerman, V. Behrana Jensen, Vidya Gopalakrishnan, Rhoneil Pena, Phi Quach, Grace Kim, Saul Kivimäe, Loui Madakamutil, Willem W Overwijk, Jonathan Zalevsky[1], Nancy Gordon

TABLE OF CONTENTS

Supplemental Fig. S1: Effect of BEMPEG treatment on ratio of effector (NK and T) cells to monocytes and to SSChi CD11b+/myeloid cells in leukocytes isolated from K7M2-WT-colonized lungs. 2

Supplementary Fig. S2: BEMPEG decreases osteosarcoma relapse to the femur. 3

Supplementary Fig. S3: Survival of mice subcutaneously implanted with DLM8 osteosarcoma tumors with combination BEMPEG and anti-CTLA-4 treatment is higher than anti-CTLA-4 alone. 4

SUPPLEMENTAL FIGURES

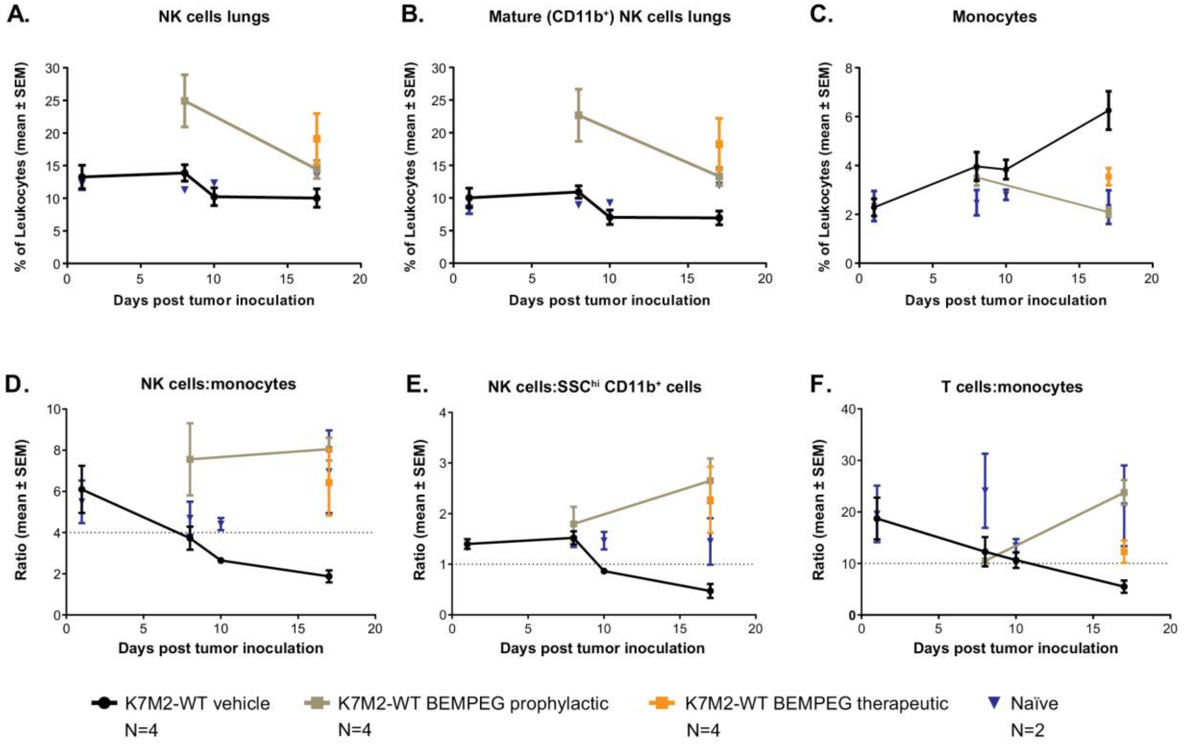

Supplemental Fig. S1: Effect of BEMPEG treatment on ratio of effector (NK and T) cells to monocytes and to SSChi CD11b+/myeloid cells in leukocytes isolated from K7M2-WT-colonized lungs. A) NK cells; B) mature (CD11b+) NK cells; C) monocytes; D) NK:monocyte ratio; E) NK:SSChi CD11b+ ratio; F) T cell:monocyte ratio.

BEMPEG, bempegaldesleukin; NK, natural killer; SEM, standard error mean.

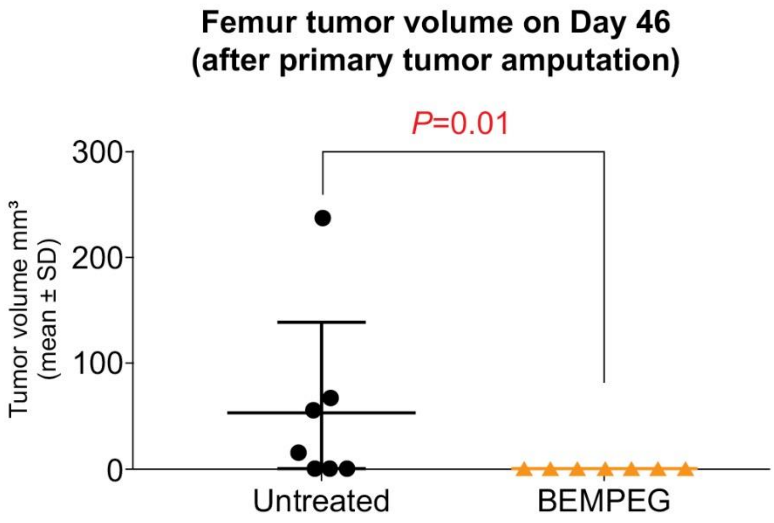

Supplementary Fig. S2: BEMPEG decreases osteosarcoma relapse to the femur. Representative histological data of mean tumor volume from animals on day 46, 13 days after amputation in the BEMPEG-treated mice vs untreated controls (P=0.01).

BEMPEG, bempegaldesleukin; SD, standard deviation.

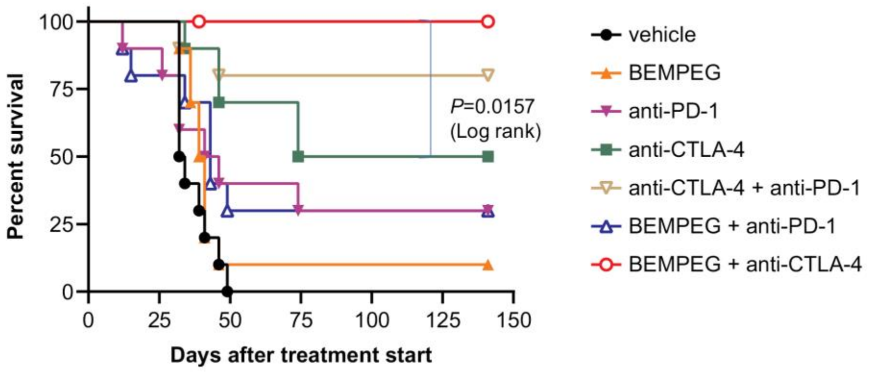

Supplementary Fig. S3: Survival of mice subcutaneously implanted with DLM8 osteosarcoma tumors with combination BEMPEG and anti-CTLA-4 treatment is higher than anti-CTLA-4 alone.

BEMPEG, bempegaldesleukin; CTLA-4, cytotoxic T-lymphocyte antigen-4;
PD-1, programmed death-1.
